# Integrative subtyping by bile acid metabolism identifies *CLCA1*/*UGT2A3*/*ZG16* as markers of immune dysfunction and poor prognosis in colorectal cancer

**DOI:** 10.3389/fonc.2025.1739534

**Published:** 2026-01-12

**Authors:** Li Feng, Min Wang, Xin Li, Long Wu, DeXin Gu, Bin Zhang, Peng Zheng, Qifeng Yang, Ke Wang, Gang Mao

**Affiliations:** 1 Department of General Surgery, Hospital of Chengdu University of Traditional Chinese Medicine, Chengdu, China; 2 Department of Plastic and Aesthetic Surgery, Hospital of Chengdu University of Traditional Chinese Medicine, Chengdu, China; 3 Department of Vascular Surgery, Hospital of Chengdu University of Traditional Chinese Medicine, Chengdu, China; 4 Gastroenterology Department, Hospital of Chengdu University of Traditional Chinese Medicine, Chengdu, China

**Keywords:** bile acid metabolism, CLCA1, colorectal cancer, tumor immune microenvironment, UGT2A3, ZG16

## Abstract

**Background:**

Colorectal cancer (CRC) is the primary driver of cancer-related death and illness across the world. Despite the full-scale shift of the treatment approach for some colorectal cancer patients due to the use of immune checkpoint inhibitors (ICIs), primary resistance still poses a huge challenge to clinicians. Bile acid metabolism is involved in the pathogenesis of CRC. However, its particular function in shaping the tumor immune microenvironment (TIME) and its effect on prognosis and immune treatment response remain unclear.

**Methods:**

Based on the transcriptome and clinical data from The Cancer Genome Atlas-Colon Adenocarcinoma (TCGA-COAD) cohort, we performed unsupervised consensus clustering and classified patients into different molecular subtypes according to bile acid metabolism. We subsequently compared overall survival (OS), immune cell infiltration levels, and differentially expressed genes among the subtypes. In addition, protein–protein interaction (PPI) network and Cox proportional hazards regression were used to identify key hub genes. Finally, the expression of these crucial hub genes was validated in the Gene Expression Omnibus (GEO) cohort and independent clinical patients.

**Results:**

The bile-low group showed a significant reduction in OS time (*p* = 0.0049). The infiltration levels of CD8^+^ T cells (*p* < 0.05) and M1 macrophages (*p* < 0.01) were significantly higher in the bile-low group than in the bile-high group. We identified three key genes—*CLCA1*, *UGT2A3*, and *ZG16*—and found that they all were downregulated in tumor tissues across the TCGA-COAD and GEO datasets, as well as in independent clinical samples. Survival analysis showed that high *CLCA1* expression was significantly associated with favorable overall survival (*p* < 0.001), whereas *UGT2A3* (*p* = 0.23) and *ZG16* (*p* = 0.17) did not reach statistical significance. The three hub genes were negatively correlated with the (TIDE) score (*CLCA1*: *R* = − 0.24, *p* < 0.001; *UGT2A3*: *R* = − 0.15, *p* = 0.0022; *ZG16*: *R* = − 0.14, *p* = 0.0039).

**Conclusion:**

Our findings suggest that bile acid metabolism could shape the TIME via key genes *CLCA1*, *UGT2A3*, and *ZG16*, and subsequently modify CRC prognosis and immunotherapy responses. These genes may serve as potential prognostic indicators and mechanistic mediators linking bile acid metabolism to T-cell dysfunction, offering insights for future combination strategies targeting the metabolism–barrier–immunity axis.

## 1 Introduction

Colorectal cancer (CRC) is a common malignant tumor worldwide. Based on the 2023 global cancer statistics, there are more than two million new cases of colorectal cancer and nearly one million cancer-related deaths per year, imposing a substantial burden on the global healthcare system (1–3).

CRC exhibits genetic instability and marked heterogeneity. The advent of immune checkpoint inhibitors (ICIs) represents a significant advance in the treatment of patients with advanced CRC and high microsatellite instability (MSI-H) (4, 5). These drugs improve patient survival by mitigating the immunosuppressive state induced by tumors (6–8). Nevertheless, the issue of “primary resistance” persists in clinical practice, and only a small proportion of patients derive benefit from ICIs (9).

Bile acids are signal-conveying molecules within the gastrointestinal tract. Beyond their essential role in lipid metabolism, bile acids also substantially influence the development of CRC, particularly when bile acid metabolism is dysregulated (10–12). Recent studies have reported that abnormal bile acid accumulation may foster tumor invasion and proliferation in CRC cells by regulating several mechanisms, including activation of nuclear receptors, induction of intestinal inflammation, and promotion of DNA damage (13–15). Previous investigations have shown that, in CRC, deoxycholic acid (DCA) remodels the tumor immune microenvironment (TIME) through synergistic mechanisms: upregulating EGFR via the PI3K/AKT pathway to influence CD8^+^ T-cell activity; inhibiting tumor protein p53 through ERK1/2 activation, thereby reducing the antitumor immune effect; and upregulating β-linked proteins to promote the proinflammatory differentiation of regulatory T cells (16).

Currently, the TIME is recognized as the most critical factor in CRC prognosis and response to ICIs (17–20). Bile acid metabolism may influence TIME by regulating immune cell infiltration and cytokine secretion (10, 11, 21), and it may also coordinate dynamic interactions among host metabolism, intestinal barrier integrity, and local immunity(metabolism–barrier–immunity axis) (22). Nevertheless, the precise mechanism linking bile acid metabolism to the tumor immune microenvironment in CRC remains unclear.

Furthermore, a systematic identification of key functional genes operating at the intersection of bile acid metabolism and tumor immune regulation is still lacking. Most prognostic models or biomarkers in CRC are not derived from dedicated analyses of this specific metabolic pathway. Therefore, it is essential to employ a pathway-centric bioinformatic strategy to unbiasedly screen for hub genes within the bile acid metabolism network, which may serve as novel prognostic indicators and reveal mechanisms of immunotherapy resistance.

**Figure 1:**
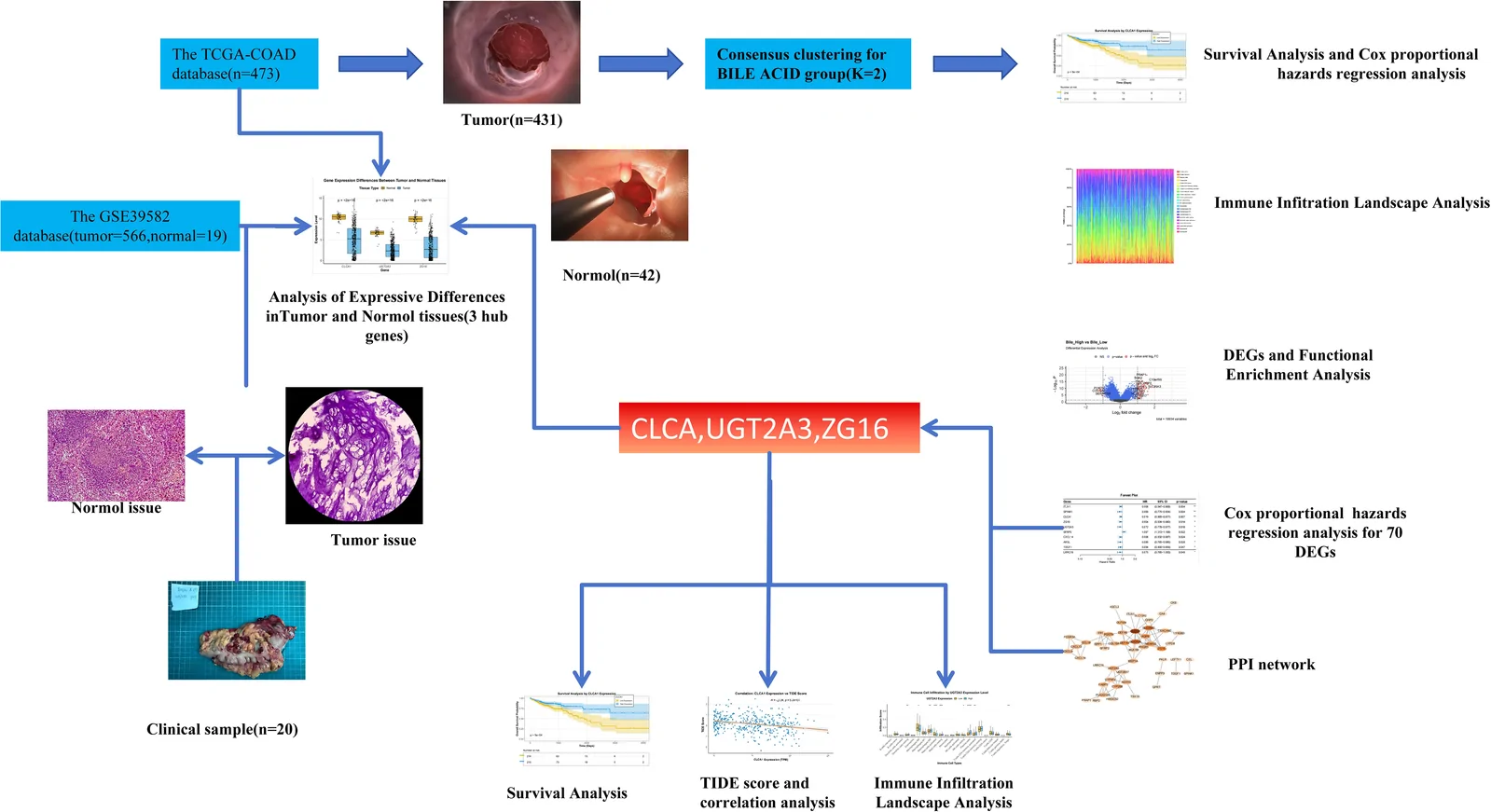
Graphical abstract.

This study aims to systematically explore the role of bile acid metabolism in shaping the CRC TIME and to identify key functional genes involved in this process using bioinformatic methods (**Figure 1**). We anticipate that the results will reveal factors linking metabolism to immune phenotypes and provide insights for developing refined combination immunotherapy strategies.

## 2 Materials and methods

### 2.1 Data source

We obtained Transcripts Per Million (TPM)-normalized RNA sequencing (RNA-seq) data for Colon Adenocarcinoma (COAD) via The Cancer Genome Atlas (TCGA) data portal (https://portal.gdc.cancer.gov/). A total of 524 samples were downloaded, comprising 455 tumor samples and 69 normal samples (23). Corresponding clinical data were retrieved from the UCSC Xena database (https://xenabrowser.net/), which initially included 530 samples. Duplicate samples were removed, and only those overlapping with the RNA-seq dataset were retained. Samples lacking clinical information were excluded, resulting in a final cohort of 480 patients, including 431 tumor samples and 49 normal samples. For validation, we obtained the GSE39582 dataset from the Gene Expression Omnibus (GEO) database (www.ncbi.nlm.nih.gov/geo/). After removing duplicates and samples with missing values, the dataset comprised 566 tumor cases and 19 normal cases. All data processing was performed using R software (v4.5.1), without applying batch correction. TCGA data were extracted and processed using the R package tidyverse (2.0.0); gene expression levels were in TPM format and were finally processed by taking the log2 + 1 value. The GSE39582 dataset was extracted and processed using the R package GEOquery (v2.76.0), and probe identifiers were converted using the corresponding GPL570–55999 platform annotation files obtained from the official GEO website.

### 2.2 Molecular subtyping based upon the bile acid metabolism pathway

We secured the gene set “HALLMARK BILE ACID METABOLISM” from the Molecular Signatures Database (MSigDB; https://www.gsea-msigdb.org/gsea/msigdb) (24), which is a curated compilation of genes specifically associated with bile acid metabolic mechanisms. Using this gene set, we calculated the bile acid metabolism gene-set score for each specimen. To identify molecular subtypes of COAD associated with bile acid metabolism, we performed unsupervised consensus clustering on filtered COAD samples via the ConsensusClusterPlus (k = 6, 1,000 resampling iterations) package (v 1.72.0) in R (25). Taking into account the above analyses and the statistical power required for subsequent survival analyses, immune infiltration comparison, and other statistical tests for each subgroup, we ultimately classified COAD patients into distinct molecular subtypes.

To assess the robustness of the molecular typing results, we used two independent Kyoto Encyclopedia of Genes and Genomes (KEGG) pathway gene sets for validation: the bile secretion pathway and the primary bile acid biosynthesis pathway (data obtained using the R package KEGGREST v1.48.1). Pathway activity scores were calculated as the average expression levels of genes within each pathway, after which correlations with the original Hallmark pathway scores were evaluated. Survival analysis was then conducted based on stratification using these gene sets. All three enrichment scores were calculated using GSVA implemented in the R package GSVA (v2.2.0).

### 2.3 Survival analysis across subgroups and Cox analyses of bile acid metabolism score

Survival and immune infiltration analyses of molecular subtypes based on bile acid metabolism were performed. Overall survival (OS) was defined as the study endpoint, and Kaplan–Meier survival curves were generated for each molecular subtype.

The log-rank (Mantel–Cox) test was used to compare survival differences among subtypes, and a two-sided *p* < 0.05 was considered statistically significant. Moreover, the connection between the continuous bile acid metabolism score and patient survival was evaluated using multivariate Cox proportional hazards regression, with results reported as hazard ratios. All statistical analyses and visualizations were conducted in R using the packages ggplot2 (v3.5.2), survminer (v0.5.0), and survival (v3.8-3).

### 2.4 Analysis of immune infiltration landscape via CIBERSORT and xCell

We employed the Cell-type Identification By Estimating Relative Subsets Of RNA Transcripts (CIBERSORT) algorithm to estimate the relative percentages of 22 immune cell types (using the LM22 signature matrix; downloaded from https://cibersort.stanford.edu/). Only samples with a deconvolution *p*-value < 0.05 from the CIBERSORT output were retained (perm = 100). The main differences in immune cell proportions among subgroups were compared using a two-sided Wilcoxon test, with *p* < 0.05 indicating statistical significance. All computations were implemented in R using the packages e1071 (v1.7-16), preprocessCore (v1.70.0), ggplot2 (v3.5.2), pheatmap (v1.0.13), and parallel (base package). To verify the stability of the above results, we further applied the xCell algorithm to 431 samples using the xCell package (v1.1.0) and then extracted the immune cell-related data for downstream analysis.

### 2.5 Sorting of differentially expressed genes and functional enrichment analysis

We identified differential expression genes (DEGs) by comparing gene expression differences across subgroups using the limma package (v3.64.3) in R. The selection criteria were |log2(fold change)| > 1 and an adjusted FDR < 0.05. KEGG enrichment analysis and Gene Ontology (GO) pathway enrichment analysis of DEGs were performed using the clusterProfiler (v4.16.0) and enrichplot (v1.28.4) R packages (26, 27).

### 2.6 Spotting and selection of hub genes

To prioritize biologically relevant and prognostically significant genes, we employed a stringent two-step filtering strategy. First, 70 DEGs were submitted to the STRING online database (28) (https://string-db.org), and a PPI network was constructed using Cytoscape software (v3.9.1). The top 20 genes with the highest degree values were selected using the “Degree” algorithm within the PPI network. Moreover, Cox analysis (R package survival, v3.8-3) was performed on the 70 identified DEGs to screen for genes significantly associated with OS, using a two-sided Wald test with *p* < 0.05 as the significance threshold (without adjustment for multiple testing). The final hub genes for validation were defined as the intersection of two gene sets.

### 2.7 Survival analysis along with immune environment analysis

Based on the screened key genes, all patients were stratified into high- and low-expression groups. Survival analysis was performed between the two subgroups. The extent of immune cell infiltration in each subgroup was analyzed using a two-sided Wilcoxon test (*p* < 0.05 was considered statistically significant). In addition, the bile-acid metabolism score (single-sample Gene Set Enrichment Analysis (ssGSEA); same method as described in Section 2.1) was compared between the two groups. Spearman correlation analysis (implemented using the stats package in R) was employed to examine correlations between gene expression levels and key T-cell chemotactic factors (C-X-C motif chemokine ligand 9 (CXCL9), C-X-C motif chemokine ligand 9 (CXCL10)). The R packages used for survival analysis and the Wilcoxon test were the same as those described previously.

### 2.8 Prediction regarding immunotherapy response

All samples’ standardized expression matrices (gene expression z-scores) were uploaded to the online Tumor Immune Dysfunction and Exclusion (TIDE) platform (link: http://tide.dfci.harvard.edu/), and relevant metrics, including the TIDE score, dysfunction score, and exclusion score, were obtained for each sample. According to the developer’s recommendations, a TIDE score > 0 indicated a predicted nonresponder, whereas a score ≤ 0 indicated a predicted responder. Differences in TIDE scores between expression-defined groups were assessed using the Wilcoxon rank-sum test, and correlations between TIDE scores and gene expression levels were evaluated using Spearman correlation analysis (29). In addition, the associations between the expression levels of *CLCA1*, *UGT2A3*, and *ZG16* and bile acid metabolism pathway activity (ssGSEA score) were examined using a two-sided Wilcoxon rank-sum test and a two-sided Spearman correlation analysis.

### 2.9 Validation of crucial gene expression patterns

#### 2.9.1 Validation of hub gene expression patterns in TCGA-COAD and GEO cohorts

To validate the differential expression of the identified hub genes, we evaluated the expression of three hub genes between tumor and normal samples in TCGA-COAD and GSE39582 datasets. A two-sided Wilcoxon rank-sum test was applied, and *p* < 0.05 was considered statistically significant.

#### 2.9.2 Testing the expression of the hub gene in clinical specimens via quantitative real-time PCR

##### 2.9.2.1 Clinical specimens

Twenty clinical samples (tumor tissues and their paired adjacent normal tissues) were collected from colon adenocarcinoma patients who underwent curative surgical resection at the Hospital of Chengdu University of Traditional Chinese Medicine. Adjacent normal tissues were obtained from tissue parts ≥ 5 cm away from the tumor margin. All specimens were stored at − 80°C for subsequent experimental analyses.

Applied Biosystems’ real-time PCR system (Foster City, CA, USA) was used to carry out qRT-PCR on all specimens. A melt-curve analysis followed the amplification profile to verify the specificity of the amplification. The 2^(−ΔΔCt) method was used to calculate the relative expression level of target genes (endogenous: glyceraldehyde-3-phosphate dehydrogenase (GAPDH)). The statistical significance of expression between tumor and normal samples was evaluated via the Wilcoxon test.

##### 2.9.2.2 Related reagents as well as consumables

The related reagents and consumables used in this study included RNA extraction solution (Cat. No. 15596-026CN, Invitrogen (Thermo Fisher Scientific, Waltham, MA, USA)); HyperScript™ III RT SuperMix for qPCR, which includes a gDNA wiper (Cat. No. K1585, APExBIO (Houston, TX, USA)); and HotStart™ 2X Green qPCR Master Mix (Cat. No. K1070, APExBIO). The primer sequences are listed as follows:

*CLCA1*: TGGTTGCTGAGTCTACTCCTC (Forward Primer); TCGTCAAATACTCCCCATCGT (Reverse Primer); *UGT2A3*: GCCTTCGTTAATTGACTACAGGA (Forward Primer); GTTGATAAGCCTGGCAAGACAT (Reverse Primer); *ZG16*: GGGTCCGAGTCAACACATACT (Forward Primer); CACCCACATAGTCGCTCCAC (Reverse Primer); GAPDH: ACAACTTTGGTATCGTGGAAGG (Forward Primer); GCCATCACGCCACAGTTTC (Reverse Primer).

The primer efficiency values ranged from 90% to 110%, with correlation coefficients (*R*^2^) ≥ 0.99, indicating high amplification specificity and reliability.

### 2.10 Statistical analysis

Continuous variables were analyzed according to the number of groups. For comparisons between two groups, the Wilcoxon rank-sum test was used; for comparisons involving more than two groups, the Kruskal–Wallis test was applied. Categorical variables were compared using the Chi-square test (*χ*^2^ test). Correlations between variables were assessed using Spearman’s rank correlation analysis. All statistical tests run were two-sided, and a *p*-value < 0.05 was considered statistically significant.

## 3 Results

### 3.1 Molecular subtyping based on bile acid metabolism highlights prognostic heterogeneity

**Figure 2:**
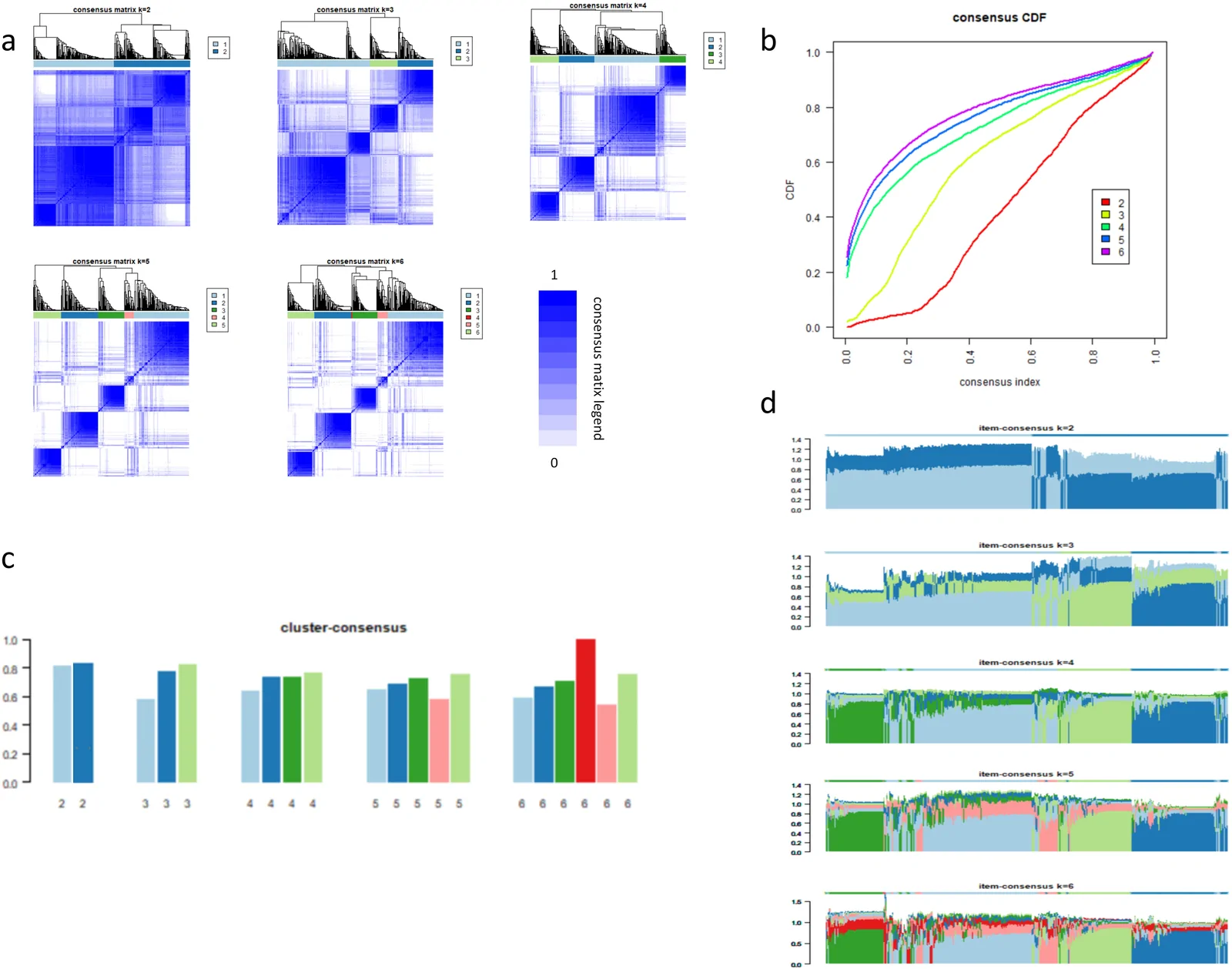
Results of consensus clustering. **(a)** Clear block structures are observed at *k* = 2, 3, and 4, whereas the matrices become fragmented and less defined at *k* = 5 and 6. Only when *k* = 2 are the sample sizes of each group relatively large (> 100 patients per group), and the consensus matrix is clearly delineated. **(b)** CDF curves show that *k* = 2 exhibits the largest separation between low and high consensus indices, suggesting strong within-cluster consensus despite potential concerns regarding overall stability. **(c)** Cluster-consensus values are relatively high for both *k* = 2 (all > 0.8) and *k* = 4; however, *k* = 2 demonstrates more stable performance. **(d)** Item-consensus analysis shows that the peak value is higher when *k* = 2, although the fluctuation is slightly greater than that observed at *k* = 4. For other values of *k*, pronounced fluctuations are evident.

**Figure 3:**
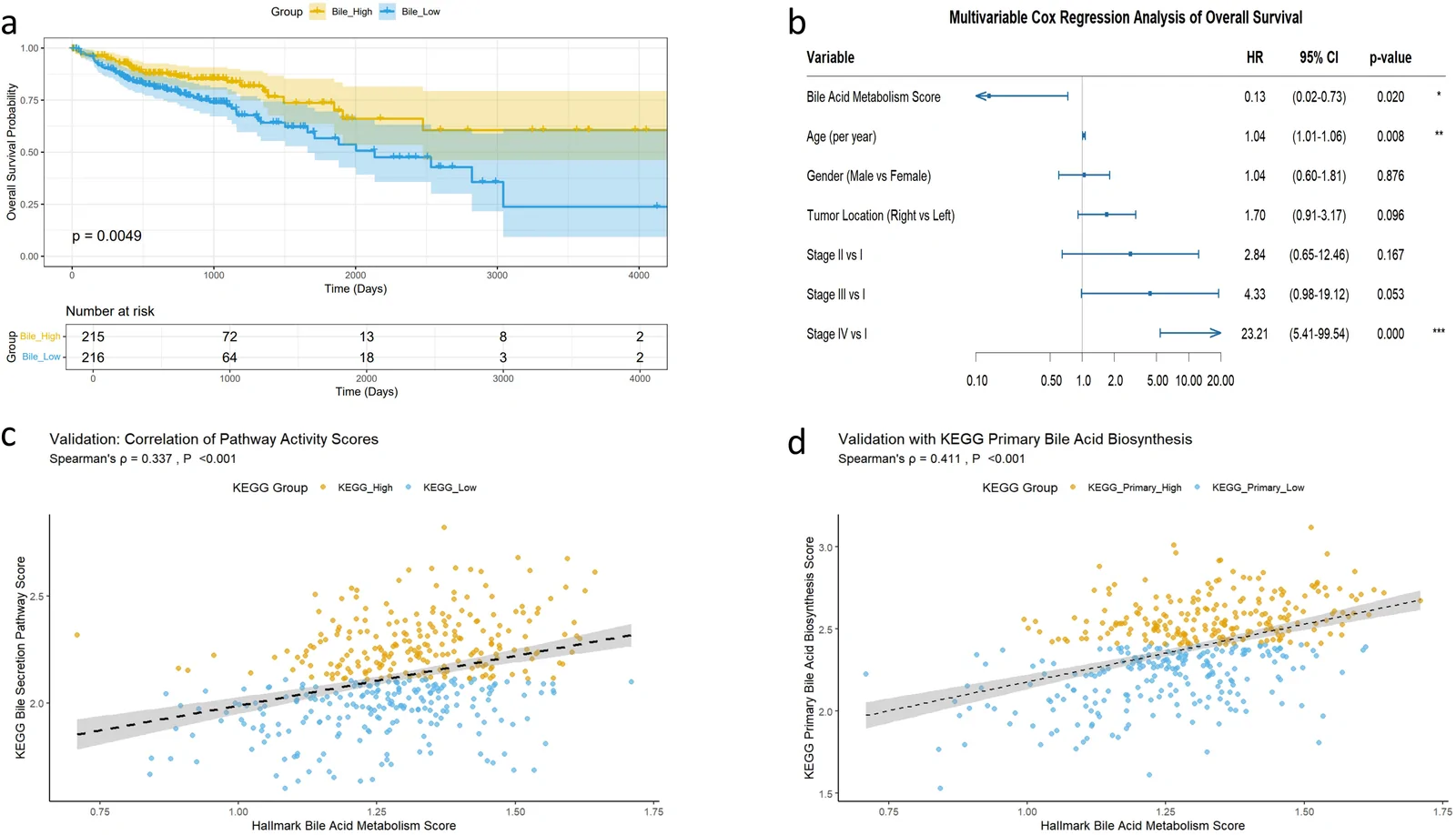
Prognostic significance and validation of the bile acid metabolism score in colorectal cancer patients. **(a)** Kaplan–Meier overall survival curves for the bile-high vs. bile-low groups (log-rank test, *p* = 0.0049). **(b)** Multivariate Cox proportional hazards model adjusted for age, gender, tumor location, and stage (HR = 0.13; 95% CI: 0.02–0.73; *p* = 0.020). **(c)** Correlation between the Hallmark bile acid metabolism score and KEGG “bile secretion” pathway scores (Spearman’s *ρ* = 0.337, *p* < 0.001). **(d)** Correlation between the Hallmark bile acid metabolism score and KEGG “primary bile acid biosynthesis” pathway scores (Spearman’s *ρ* = 0.411, *p* < 0.001).

To establish a molecular subtyping system based on bile acid metabolism, we applied unsupervised consensus clustering to evaluate clustering schemes with *k* ranging from 2 to 6. As *k* increased, the diagonal of the consensus matrix became progressively clearer (**Figure 2a**). The CDF curve exhibited a distinct separation and clear upward trend at *k* = 2 (**Figure 2b**), while both cluster-consensus and item-consensus analyses indicated that *k* = 2 and *k* = 4 had the highest stability (**Figures 2c, d**). Although *k* = 4 demonstrated comparable stability, *k* = 2 provided the advantage of more balanced subgroup sizes (> 100 patients per group), ensuring sufficient statistical power for downstream survival and immune analyses and avoiding potential overstratification. When *k* = 2, the patients were divided into two subgroups with balanced sample sizes (bile-high group, *n* = 215; bile-low group, *n* = 216), which enhanced the statistical power of subsequent analyses. The baseline characteristics of the two subgroups are presented in **Table 1**. No significant differences were observed in age (*p* = 0.774, Wilcoxon test), gender (*p* = 0.598, Chi-square test), and tumor stage (*p* = 0.292, Chi-square test) between the groups. However, tumor location differed significantly between the subgroups (*p* = 0.001, Chi-square test). Therefore, this grouping scheme was adopted for further analyses. Survival analysis indicated that overall survival was significantly shorter in the bile-low group compared with the bile-high group (*p* = 0.0049, **Figure 3a**). Multivariate Cox regression analysis, adjusting for key clinical confounding factors, confirmed that this subtyping model was an independent prognostic factor (HR = 0.13; 95% CI: 0.02–0.73; *p* = 0.020; **Figure 3b**). Independent validation analysis demonstrated that the activity scores of the KEGG bile secretion pathway and the primary bile acid biosynthesis pathway were significantly positively correlated with the original Hallmark bile acid metabolism score (**Figures 3c, d**; Spearman’s *ρ* = 0.337 and 0.411, respectively; *p* < 0.001 for both).

**Table 1:** Baseline characteristics of patients in the bile-high and bile-low groups.

Group	Bile high (*n* = 215)	Bile low (*n* = 216)	*p*-value
Age (year, mean ± SD)	66.2 ± 13	66.7 ± 12.6	0.774
Gender (man)	113 (52.6%)	119 (55.1%)	0.598
Pathological stage (cases)			
Stage I	33 (19.4%)	33 (18.5%)	0.292
Stage II	77 (45.3%)	65 (36.5%)	
Stage III	41 (24.1%)	56 (31.5%)	
Stage IV	19 (11.2%)	24 (13.5%)	
Unknown	45 (20.9%)	38 (17.5%)	
Tumor location (cases)			
Left sided	81 (37.6%)	54 (20.5%)	0.001
Right sided	83 (38.6%)	120 (55.5%)	
Unknown	51 (23.7%)	42 (19.4%)	

### 3.2 Two nonidentical subtypes possess different tumor immune microenvironment profiles

**Figure 4:**
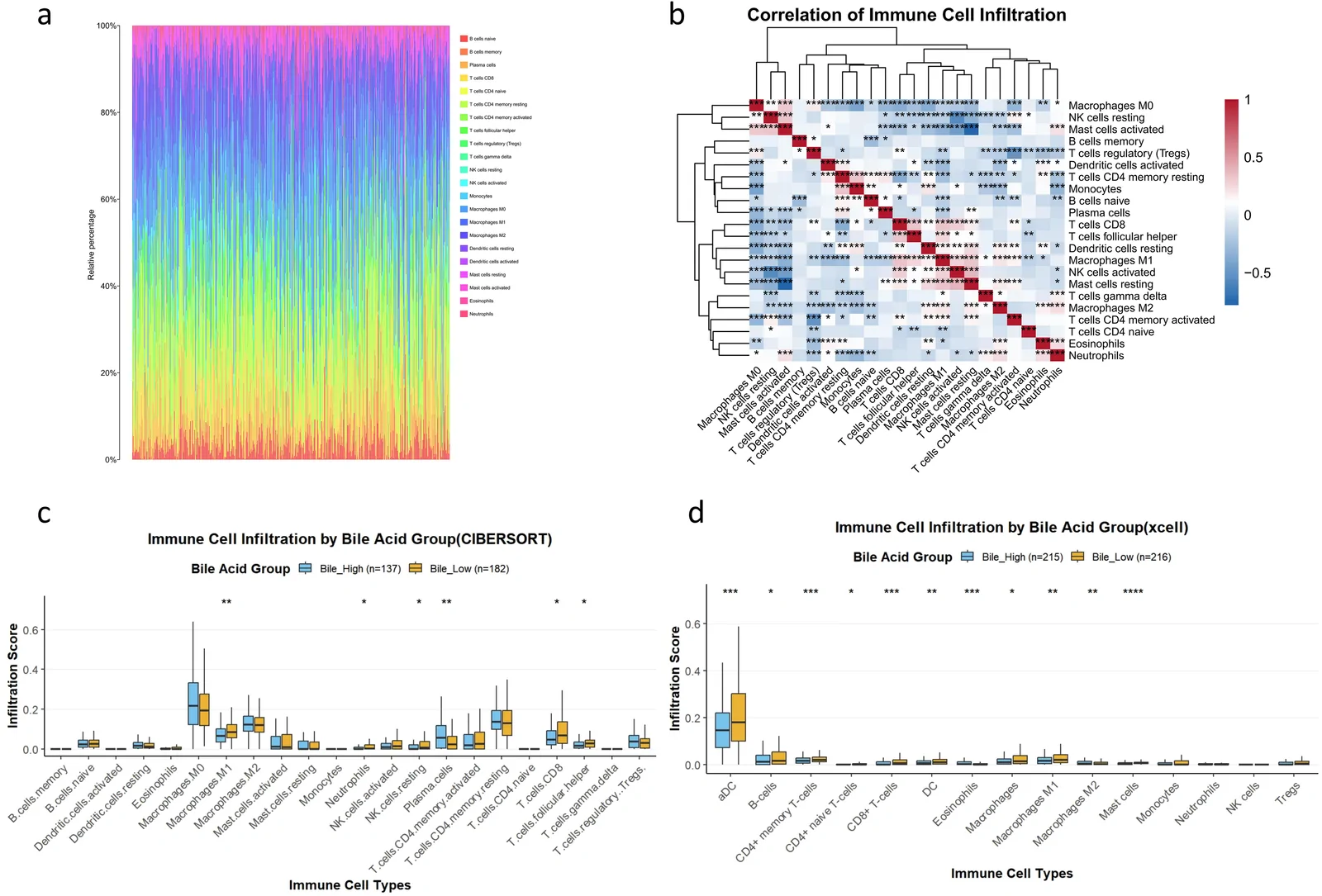
Immune cell infiltration landscape. **(a)** Heatmap of immune cell infiltration scores estimated using CIBERSORT across all samples (*n* = 319). **(b)** Correlation heatmap of immune cell proportions (Spearman’s correlation; ^*^p < 0.05, ^**^p < 0.01, ^***^p < 0.001). **(c)** Comparison of immune infiltration between the bile-high (*n* = 137) and bile-low (*n* = 182) groups using CIBERSORT (Mann–Whitney *U* test; ^*^p < 0.05, ^**^p < 0.01). **(d)** Immune infiltration comparison using the xCell algorithm (bile-high group: *n* = 215; bile-low group: *n* = 216; Mann–Whitney *U* test; ^*^p < 0.05, ^**^p < 0.01, ^***^p < 0.001).

Based on the results of CIBERSORT, a total of 319 samples (74% for total samples) had *p* < 0.05 and were included in the subsequent immune infiltration analysis. We examined the immune cell infiltration landscape of these samples (**Figure 4a**). Correlation analysis revealed that cytotoxic lymphocytes (CD8^+^ T cells and NK cells) were significantly positively correlated (**Figure 4b**), which may play an important role in antitumor immunity. Interestingly, the abundance of CD8^+^ T cells was negatively correlated with the abundance of immunosuppressive cells, suggesting a potential antagonistic relationship between effector and suppressive immune components. **Figure 4c** depicts the detailed distribution of specific immune cell types between the two groups (CIBERSORT). Compared with the bile-high group, the bile-low group exhibited significantly higher levels of CD8^+^ T cells and M1 macrophages (*p* < 0.05, **Figure 4c**). In addition, the xCell analysis confirmed that, in addition to the consistent infiltration trends of CD8^+^ T cells (*p* < 0.001) and M1 macrophages (*p* < 0.01) observed in the CIBERSORT results, B cells (*p* < 0.05) and CD4^+^ naive T cells (*p* < 0.05) also showed increased infiltration in the bile-low group compared with the bile-high group (**Figure 4d**). These findings provide important information for further exploring the mechanisms by which bile acid metabolism regulates the function of specific immune cell types.

### 3.3 Sorting out differentially expressed genes and functional enrichment scan

**Figure 5:**
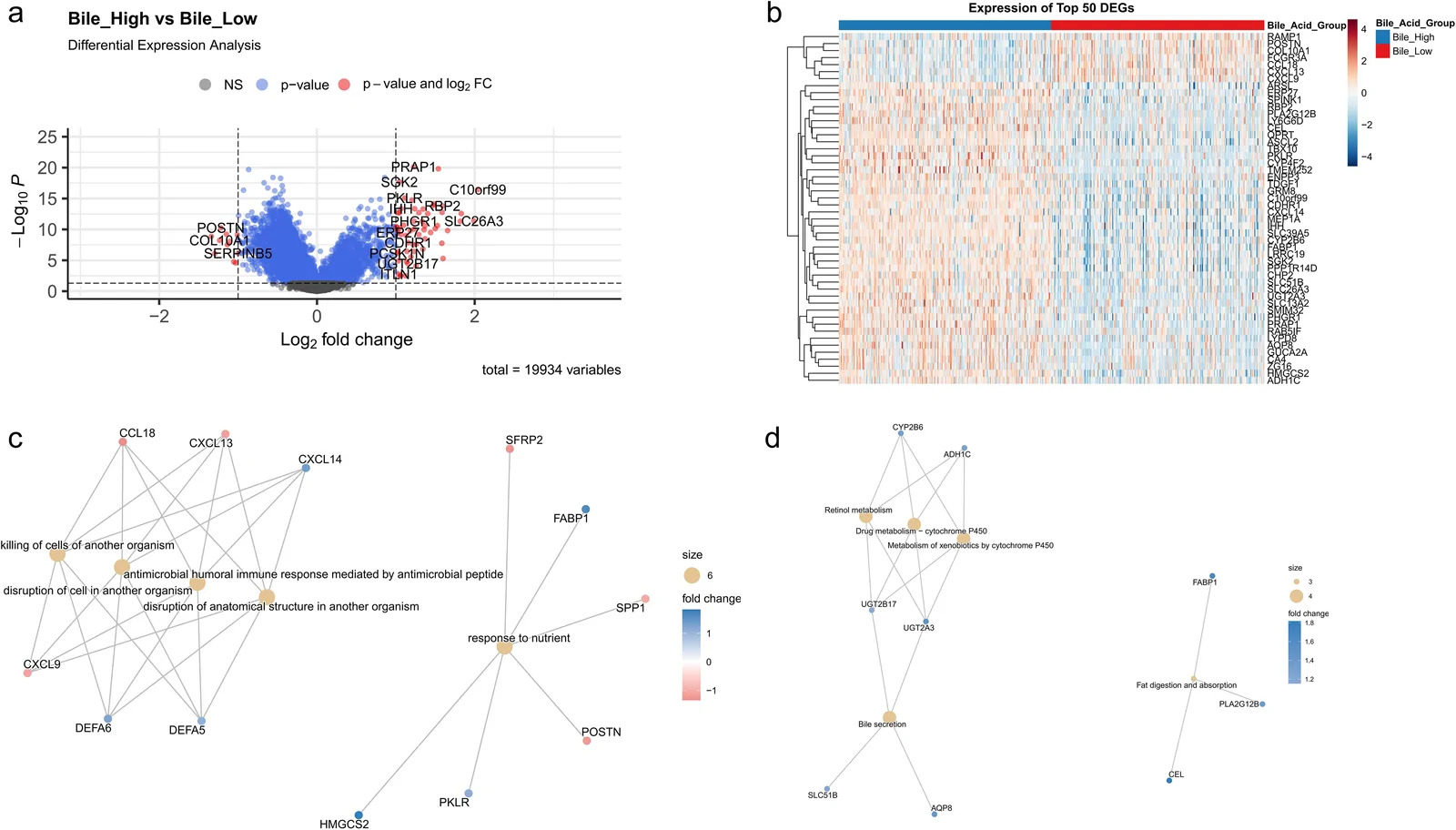
Differentially expressed genes (DEGs) and functional enrichment analysis. **(a)** Volcano plot showing DEGs (*n* = 19,934). Red dots indicate significant upregulation (adjusted *p* < 0.05, |log_2_ FC| > 1), blue dots indicate significant downregulation, and gray dots indicate nonsignificant genes. **(b)** Heatmap of the top 50 DEGs clustered by expression pattern (*z*-score normalized), with samples grouped according to bile acid metabolism status. **(c)** GO enrichment network of upregulated DEGs (size: gene count; color: log_2_ fold change). **(d)** KEGG enrichment network of downregulated DEGs (same scale as **c**).

Intergroup comparison identified 70 significantly DEGs, of which 59 were upregulated, and 11 were downregulated. The distribution of these genes is illustrated in a volcano plot (**Figure 5a**), while a heatmap in **Figure 5b** shows the top 50 genes. GO analysis indicated that the DEGs were primarily associated with the biological processes “killing of cells of other organisms,” “antimicrobial peptide-mediated humoral immune response,” and “response to nutrients” (**Figure 5c**). KEGG pathway analysis revealed that the DEGs were notably enriched in “retinol metabolism,” “drug metabolism–cytochrome P450,” and “fat digestion and absorption” (**Figure 5d**).

### 3.4 Singling out hub genes *CLCA1*, *UGT2A3*, and *ZG16*

**Figure 6:**
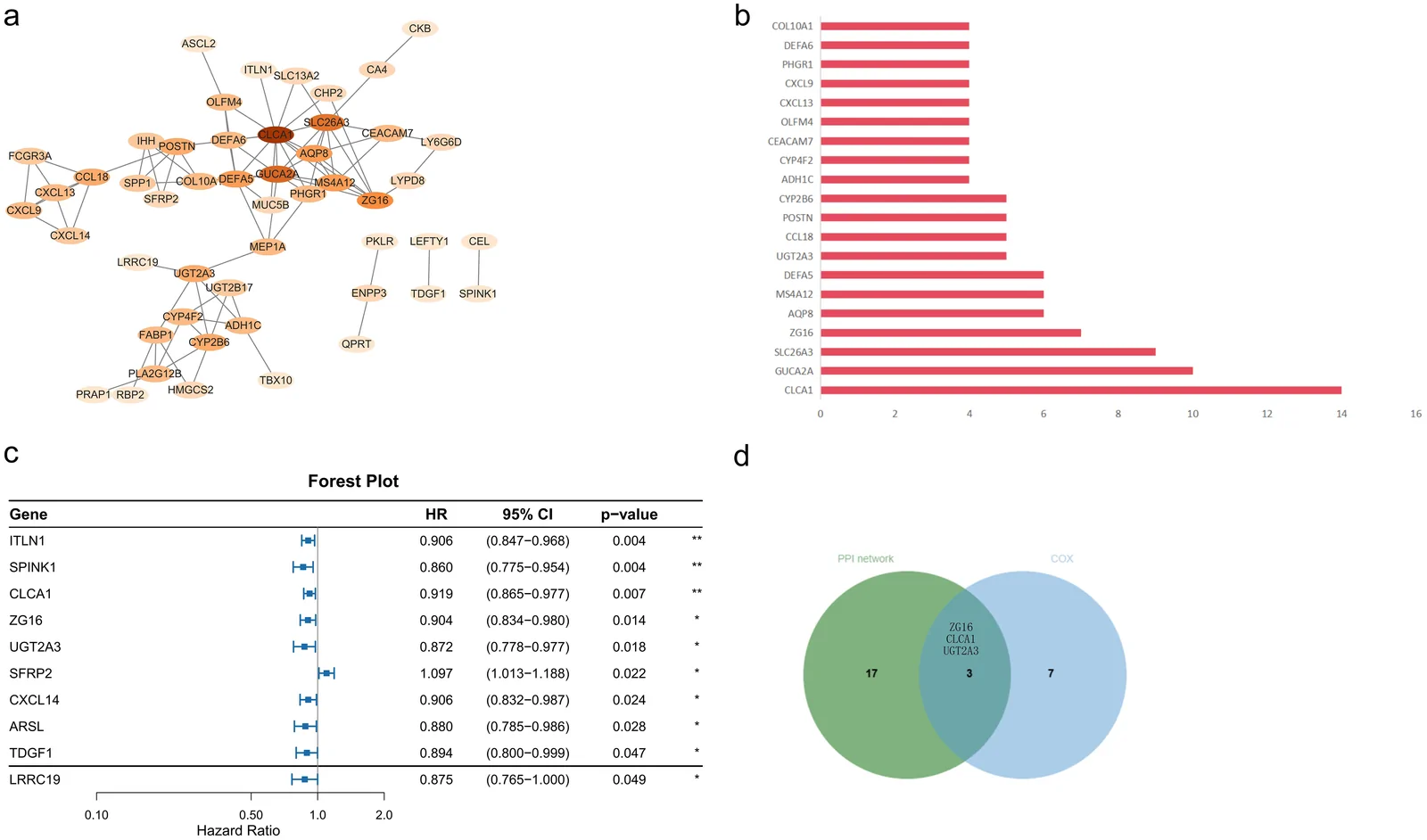
Identification of hub genes. **(a)** PPI network constructed from differentially expressed genes, in which nodes represent genes and edges indicate interactions; central hub genes are highlighted. **(b)** Top 20 hub genes ranked by degree within the PPI network. **(c)** Forest plot of univariate Cox regression analysis for candidate hub genes (HR < 1 indicates a favorable prognosis; ^*^p < 0.05, ^**^p < 0.01). **(d)** Venn diagram showing the overlap between hub genes (PPI network) and significant prognostic genes (Cox analysis); three genes (*ZG16*, *CLCA1*, *UGT2A3*) are shared between the two sets.

Subsequently, the top 20 hub genes with the highest degree values were selected from the PPI network (**Figures 6a, b**). Univariate Cox regression analysis revealed 10 DEGs significantly associated with OS (*p* < 0.05; **Figure 6c**). Given that the Cox regression analysis was not adjusted for multiple testing, these genes were considered exploratory candidate prognostic genes. The intersection of hub genes from the PPI network and DEGs significantly correlated with OS yielded three genes: *ZG16*, *CLCA1*, and *UGT2A3* (**Figure 6d**).

### 3.5 Prognostic value as well as immunomodulatory effects of hub genes

**Figure 7:**
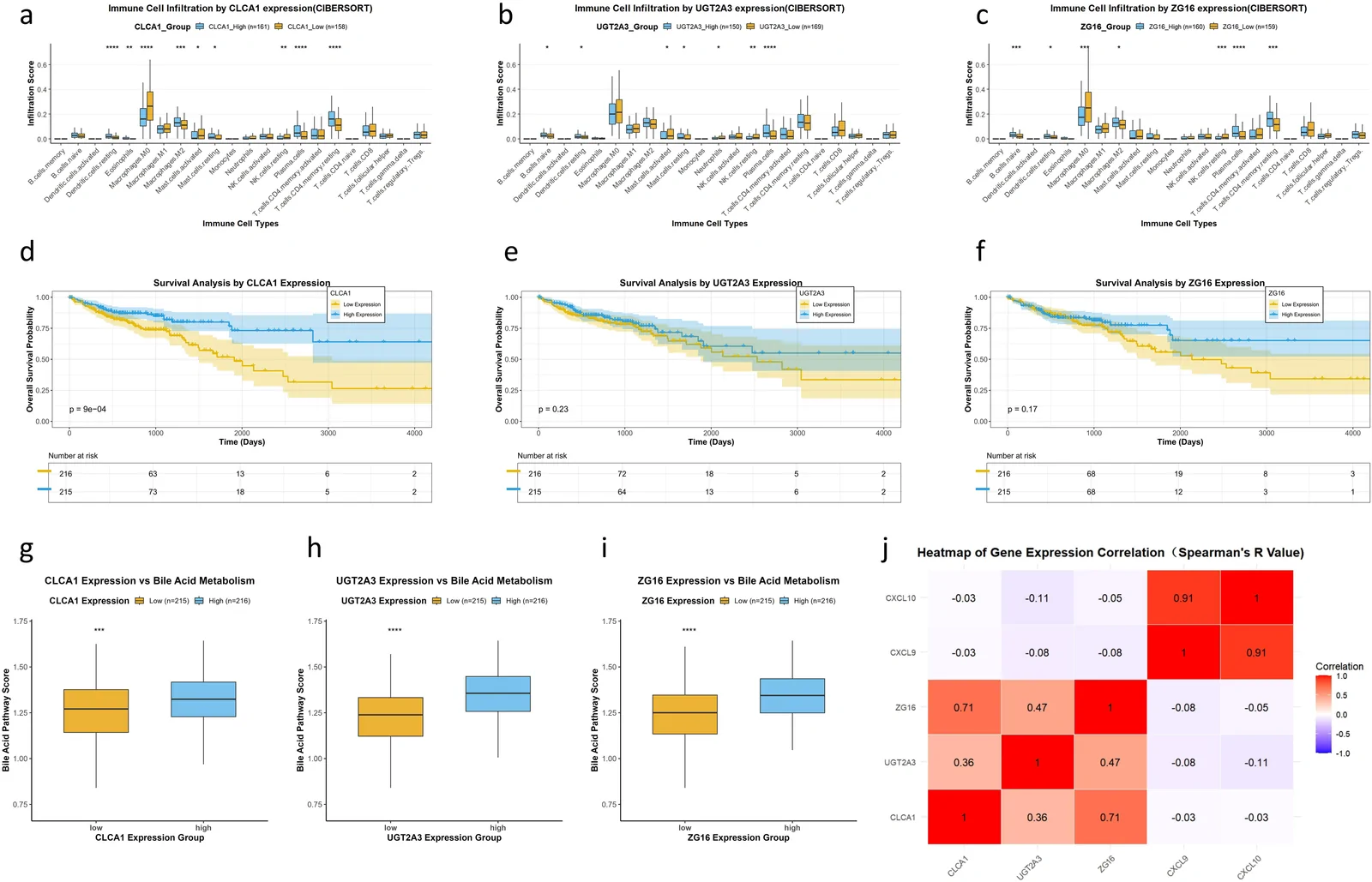
Functional and prognostic validation of the hub genes *CLCA1*, *UGT2A3*, and *ZG16*. **(a–c)** Immune cell infiltration profiles estimated by CIBERSORT in high- vs. low-expression groups of *CLCA1* **(a)**, *UGT2A3* **(b)**, and *ZG16* **(c)** (Mann–Whitney *U* test; ^*^p < 0.05, ^**^p < 0.01, ***p <0.001, ****p <0.0001). **(d–f)** Kaplan–Meier survival curves stratified by expression levels of *CLCA1* **(d)**, *UGT2A3* **(e)**, and *ZG16* **(f)** expression levels (log-rank test). **(g–i)** Bile acid metabolism pathway scores in low- vs. high-expression groups of each gene (Mann–Whitney *U* test; ^**^p < 0.01, ^***^p < 0.001, ****p < 0.0001). **(j)** Heatmap of Spearman correlations between the three hub genes and key immune-related genes (CXCL9, CXCL10); red indicates positive correlation, and purple indicates negative correlation.

Regarding immunocyte infiltration, *CLCA1* expression was associated with distinct infiltration patterns of various immune cell types compared with low expression (**Figure 7a**). Similarly, high expression of *UGT2A3* and *ZG16* showed significantly different infiltration scores across multiple immune cell types compared with low expression (**Figures 7b, c**). Survival analysis indicated that patients with high *CLCA1* expression had notably better overall survival than those with low expression (*p* = 9*e*−04; **Figure 7d**). Although the survival curves for *UGT2A3* (*p* = 0.23; **Figure 7e**) and *ZG16* (*p* = 0.17; **Figure 7f**) appeared visually separated, the differences did not reach statistical significance; these genes should therefore be considered hypothesis-generating candidates requiring further validation. Notably, patients with high expression of *CLCA1*, *UGT2A3*, or *ZG16* also exhibited significantly higher bile acid metabolism scores (all *p* < 0.001; **Figures 7g–i**), confirming that these hub genes are functionally linked to bile acid metabolic activity in CRC. Spearman correlation analysis revealed that the expression of these three genes was not significantly associated with CXCL9 or CXCL10 expression (all Spearman’s correlation coefficients |*R*| < 0.19; **Figure 7j**).

### 3.6 A low-expression degree of hub genes is associated with immunotherapy resistance

**Figure 8:**
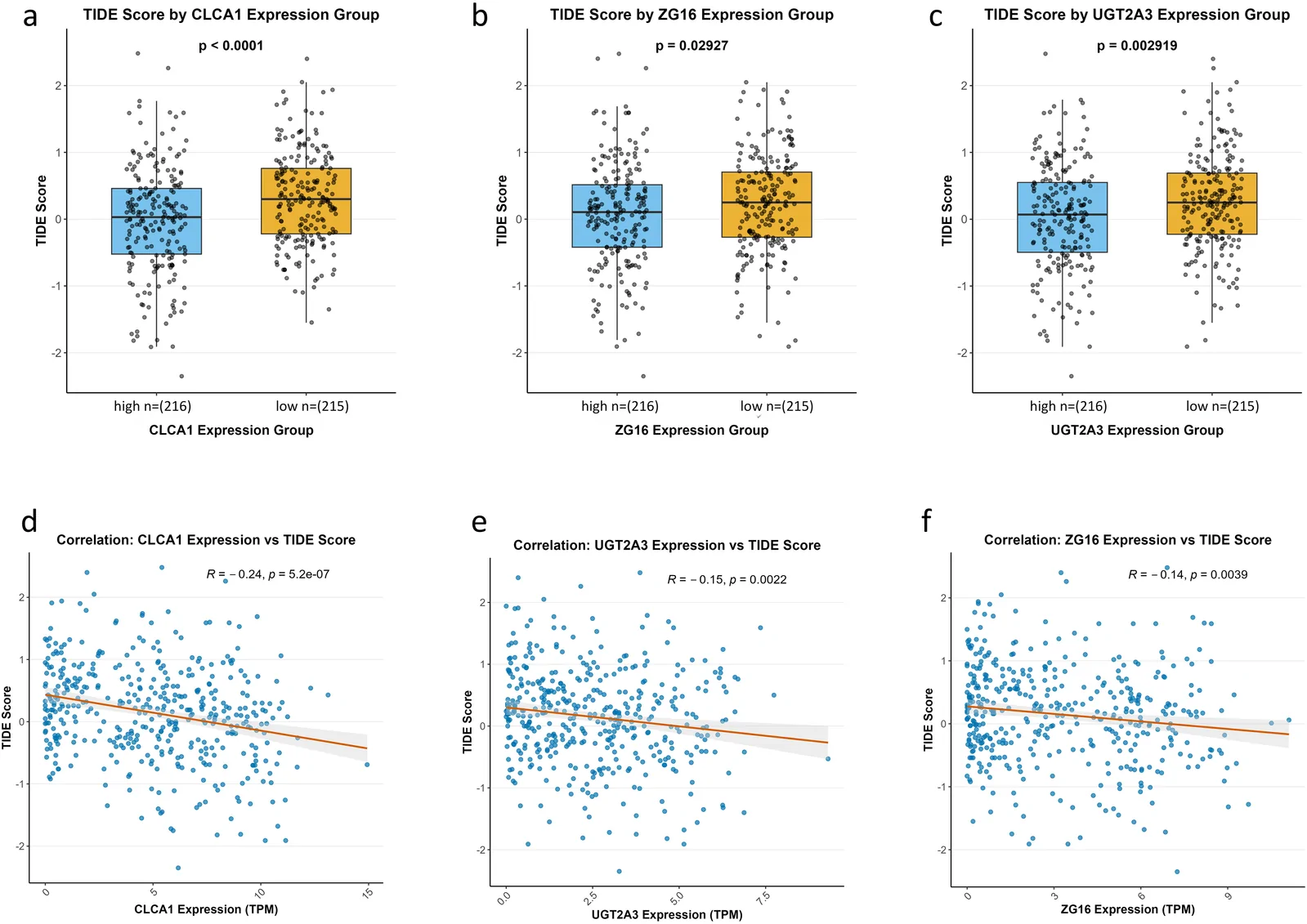
Association between hub gene expression and TIDE immunotherapy response score. **(a–c)** Box plots showing TIDE scores in high- vs. low-expression groups of *CLCA1* **(a)**, *ZG16* **(b)**, and *UGT2A3* **(c)** (Wilcoxon rank-sum test; *p*-values shown). **(d–f)** Scatter plots showing correlations between gene expression (TPM) and TIDE score: *CLCA1* **(d)** (*R* = − 0.24, *p* = 5.2 × 10^−7^), *UGT2A3* **(e)** (*R* = − 0.15, *p* = 0.0022), and *ZG16* **(f)** (*R* = − 0.14, *p* = 0.0039); linear regression lines with 95% confidence intervals are shown.

TIDE analysis indicated that the low-expression groups of *CLCA1*, *UGT2A3*, and *ZG16* had significantly higher TIDE scores compared with the high-expression groups (*p* < 0.0001, *p* = 0.0029, *p* = 0.029; **Figures 8a–c**). Remarkably, Spearman correlation analysis revealed a significant negative correlation between the expression of these three genes and the TIDE score (*CLCA1*: *R* = − 0.24, *p* < 0.001; *UGT2A3*: *R* = − 0.15, *p* = 0.0022; *ZG16*: *R* = − 0.14, *p* = 0.0039; **Figures 8d–f**).

### 3.7 Expression validation of crucial genes in TCGA database and clinical patients

#### 3.7.1 Expression in TCGA-COAD and GEO patient cohorts

**Figure 9:**
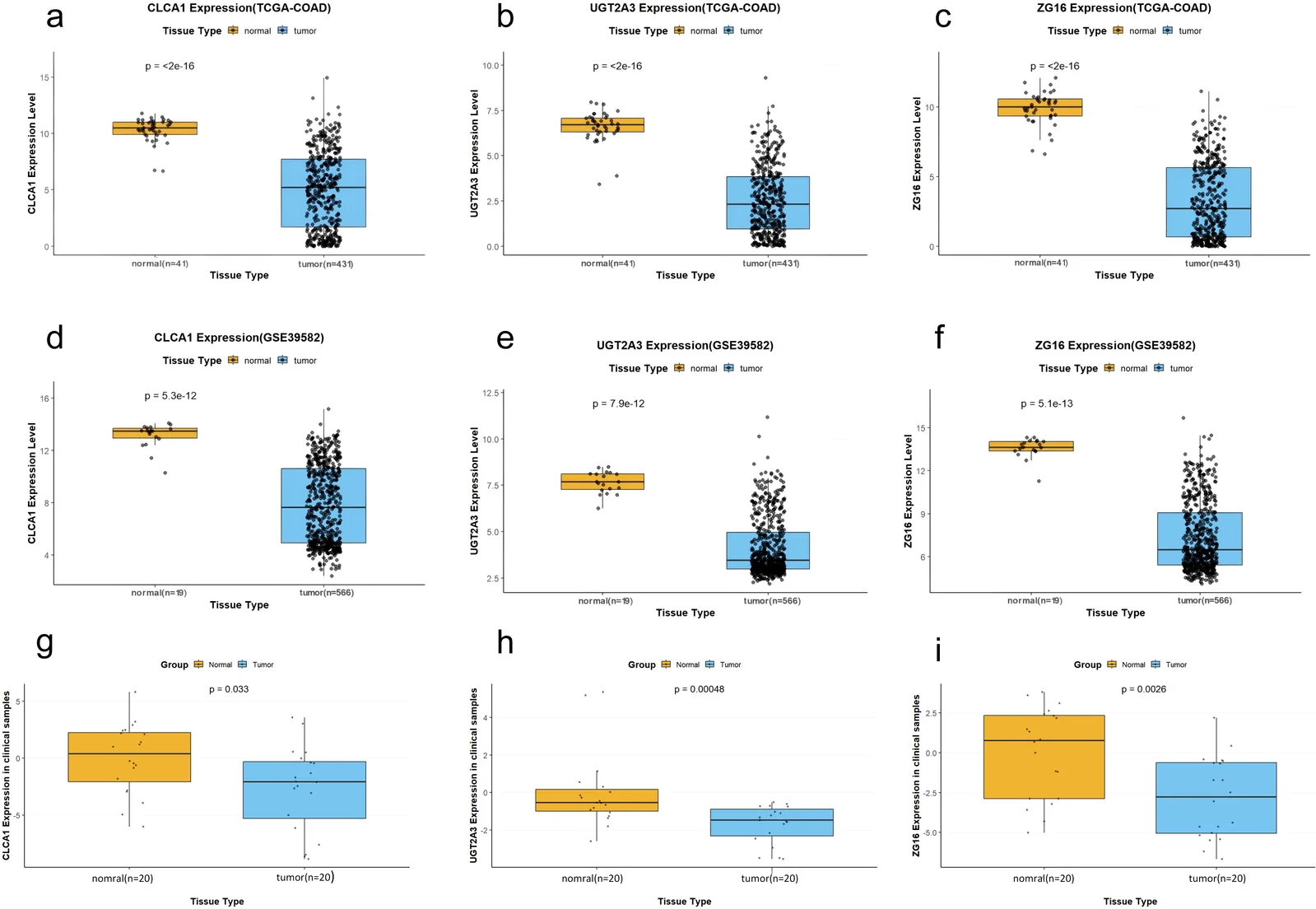
Downregulation of *CLCA1*, *UGT2A3*, and *ZG16* in colorectal cancer tissues across multiple datasets. **(a–c)** Expression levels of *CLCA1* **(a)**, *UGT2A3* **(b)**, and *ZG16* **(c)** in normal (*n* = 41) vs. tumor (*n* = 431) tissues from the TCGA-COAD cohort (Wilcoxon rank-sum test; *p* < 2 × 10^−16^). **(e–g)** Validation in the GSE39582 dataset (*n* = 19 vs. 566): *CLCA1* **(d)**, *UGT2A3* **(e)**, and *ZG16* **(f)** (all *p* < 1 × 10^−12^). **(g–i)** Expression in paired clinical samples (*n* = 20 per group): *CLCA1* **(g)** (*p* = 0.033), *UGT2A3* **(h)** (*p* = 0.00048), and *ZG16* **(i)** (*p* = 0.0026) (Wilcoxon rank-sum test).

In TCGA-COAD cohort (41 normal, 431 tumor patients), all three hub genes were significantly downregulated in tumor specimens (All *p* < 0.001; **Figures 9a–c**). A similar pattern was observed in the GSE39582 dataset (19 normal and 566 tumor patients), where the expression of all three genes was also significantly downregulated in tumor tissues (All *p* < 0.001; **Figures 9d–f**).

#### 3.7.2 Clinical independent patients’ expression

We examined a total of 20 pairs of clinical samples; the basic information of the patients is presented in **Table 2**. Normal tissues showed significantly higher relative expression of *CLCA1*, *UGT2A3*, and *ZG16* compared with tumor tissues. All three genes were notably downregulated in tumor tissues relative to adjacent normal tissues (All *p* < 0.05; **Figures 9g–i**), consistent with results from TCGA and GEO databases.

**Table 2:** Clinical sample basic information.

Characteristic	Patients (*n* = 20)
Age (year, mean ± SD)	61.45 ± 12.39
Gender (man)	16 (80%)
Pathological stage (patients)	
Stage I	2 (10%)
Stage II	8 (40%)
Stage III	9 (45%)
Stage IV	1 (5%)
Tumor location (patients)	
Left sided	13 (65%)
Right sided	7 (35%)

## 4 Discussion

In this study, we investigated the bile acid metabolism in CRC through comprehensive bioinformatics analyses and clinical patient validation. Our findings indicate that bile acid metabolism can modulate the TIME in CRC. Furthermore, three genes (*CLCA1*, *UGT2A3*, and *ZG16*) were significantly downregulated in tumor tissues. Low expression of these three genes was potentially associated with poor prognosis and diminished response to immunotherapy. These findings further support the potential vital role of bile acid metabolism in immune regulation and highlight candidate biomarkers for the future development of personalized immunotherapy strategies.

### 4.1 Correlation and conceivable mechanisms: bile acid metabolism and immune microenvironment

Our study reveals a paradoxical immune landscape in the bile-low subgroup: despite elevated infiltration of CD8^+^ T cells and M1 macrophages, patients exhibit significantly worse survival. This suggests that immune cell quantity alone is insufficient for effective antitumor immunity, and functional impairment may predominate. Notably, we found no significant correlation between *CLCA1*/*UGT2A3*/*ZG16* expression and key T-cell chemokines CXCL9/CXCL10, implying that the observed T-cell accumulation is unlikely to be driven by canonical chemotactic signaling. Instead, it may reflect T-cell exhaustion or spatial exclusion—a phenotype captured by the TIDE score but not by bulk infiltration metrics. The strong association of low bile acid metabolism with right-sided tumors further suggests site-specific metabolic-immune crosstalk, possibly mediated by microbiota or bile acid gradients (16, 30–33).

### 4.2 *CLCA1*, *UGT2A3*, and *ZG16* present a link between metabolism, mucosal immunity, and immunotherapy

A notable finding of this study is that *CLCA1*, *UGT2A3*, and *ZG16*—three genes highly expressed in normal colorectal tissues—are consistently downregulated in tumor tissues, suggesting that their loss may represent a coordinated event in CRC progression. Low expression of these genes is significantly associated with higher TIDE scores, indicative of immunotherapy resistance. Among the hub genes, only *CLCA1* exhibited a robust association with favorable overall survival, whereas *UGT2A3* and *ZG16* serve as hypothesis-generating candidates that warrant further validation. Importantly, these hub genes also display strong positive correlations with bile acid metabolism pathway activity, highlighting their potential role as functional links between metabolic dysregulation and immune dysfunction in colorectal cancer.

*CLCA1* is an accessory protein for chloride ion channels and plays a well-established role in mucus secretion and the maintenance of intestinal epithelial barrier integrity. Reduced expression of *CLCA1* may cause “damage to the intestinal mucosal barrier,” increasing local inflammation and attracting immunosuppressive cells, which can facilitate tumor development (34). Based on our findings, we hypothesize that *CLCA1* loss in CRC not only impairs the physical barrier but also generates proinflammatory milieu that attracts immunosuppressive cells. Consistent with this, lower *CLCA1* expression was associated with higher TIDE scores, suggesting that *CLCA1* deficiency contributes to a TIME characterized by T-cell dysfunction, potentially due to the failure to maintain a mucosal microenvironment supportive of effective immunity.

*UGT2A3* functions as an essential second-stage enzyme in bile acid metabolism, catalyzing the glucuronidation of bile acids to facilitate their excretion. Reduced expression of *UGT2A3* may lead to the “accumulation of toxic bile acids within cells, activation of oncogenic signaling pathways, and induction of endoplasmic reticulum stress. This, in turn, could recruit immunosuppressive cell populations through the release of specific chemokines, further remodeling the TIME (35). In this study, *UGT2A3* expression was significantly downregulated in tumors. We hypothesize that decreased *UGT2A3* expression may result in the accumulation of specific bile acid species, triggering stress-response or protumorigenic signaling pathways. The precise mechanisms underlying these effects require further investigation in future studies.

*ZG16* is a lectin, and its reduced expression may impair host–microbiota interactions, increase the risk of pathogenic bacteria translocation, trigger chronic inflammation, and create an immunosuppressive environment conducive to tumor growth (36). We speculate that decreased *ZG16* expression in CRC could destabilize the selective interface with the gut microbiota, facilitating the translocation of pathogenic bacteria or altering microbially derived signals, thereby promoting a state of chronic, low-grade inflammation.

All three genes illustrate how the interruption of the “metabolism–barrier–immunity” axis contributes to colorectal cancer progression and T-cell dysfunction, offering a new perspective for exploring the mechanisms of immune escape in CRC. Naturally, this hypothesis requires further validation.

### 4.3 Clinical translation viability: from biomarkers to combination therapy targets

The observed association between low *CLCA1*, *UGT2A3*, and *ZG16* expression and elevated TIDE scores highlights a potential link between bile acid metabolism and T-cell dysfunction in CRC. The consistency with TIDE (a tool specifically focused on T-cell exhaustion and exclusion) suggests that these genes may play a role in functional immune impairment. From another perspective, the TIDE score is designed to model T-cell dysfunction and immune rejection and is particularly effective for predicting responses to ICIs. This indicates that these genes may influence the functional state of T cells rather than overall immune infiltration or cytotoxic potential. This interpretation aligns with the phenotype observed in our study, characterized by “high CD8^+^ T-cell infiltration but poor prognosis”. Thus, rather than functioning as standalone predictive biomarkers, *CLCA1*, *UGT2A3*, and *ZG16* are more likely mechanistic mediators linking bile acid metabolism to T-cell dysfunction in CRC. Animal experiments have confirmed that a circular single-stranded DNA (CSSD) can upregulate *CLCA1* expression (37). Similarly, the *UGT2A3* expression can be modulated using specific antibodies (38). Additionally, interventions targeting the intestinal microbiota can indirectly upregulate the expression of genes like *ZG16*, thereby reshaping the TIME (39). However, studies investigating the regulation of these three key genes remain at the preclinical stage or have been conducted in other tumor types.

### 4.4 The limitations of our study

1. The design of this study was primarily retrospective. Although we verified our results using an independent clinical cohort, the relatively small sample size may pose certain limitations. To further assess the prognostic and predictive clinical relevance of these genes, large prospective cohort studies will be required.
2. We initially observed associations between the expression levels of these three genes and TIME. However, the definite molecular mechanisms through which these genes influence the TIME remain largely unknown. In future work, we plan to perform functional studies (using gene-edited cell lines, patient-derived organoids, and *in vivo* mouse models) to elucidate how these genes modulate T-cell activation and macrophage polarization.
3. Given the well-established crosstalk between the gut microbiota and bile acid metabolism, the absence of microbiome data represents a limitation of this research. Future investigations assessing microbial composition could provide crucial insights into the mechanism by which the microbiota–bile acid axis regulates the TIME.
4. Although the TIDE score can provide insights into T-cell function, it cannot yet serve as a direct evaluation tool for immunotherapy in CRC. The study also lacks clinical data related to immunotherapy. The mechanisms by which the three hub genes influence T-cell function are inferred solely from correlation between their expression levels and the TIDE score, as well as their known biological roles; experimental validation is required to confirm these effects. Additionally, the absence of MSI and microbial data limits our ability to dissect these interactions, representing an important study limitation.
5. Although *k* = 4 demonstrated comparable stability in consensus clustering, we selected *k* = 2 to prioritize clinical translatability by creating clearly dichotomized subgroups—a trade-off between granularity and practical utility.

## 5 Conclusion

In conclusion, our study demonstrates that bile acid metabolism contributes to the modulation of the immunosuppressive microenvironment in CRC and highlights *CLCA1*, *UGT2A3*, and *ZG16* as candidate molecules mediating this process. These genes may serve as potential prognostic biomarkers and mechanistic links between bile acid metabolism and T-cell dysfunction, providing insights for future combination strategies targeting the metabolism–barrier–immunity axis.

## Data Availability

The Cancer Genome Atlas (TCGA)-COAD dataset used in this study is available from TCGA data portal (https://portal.gdc.cancer.gov/) and the Gene Expression Omnibus (GEO) database (www.ncbi.nlm.nih.gov/geo/).

## References

[1] Bray F Laversanne M Sung H Ferlay J Siegel R. L Soerjomataram I . Global cancer statistics 2022: GLOBOCAN estimates of incidence and mortality worldwide for 36 cancers in 185 countries. CA-A Cancer J Clin. (2024) 74:229–63. doi: 10.3322/caac.21834 38572751

[2] Jokhadze N Das A Dizon DS . Global cancer statistics: A healthy population relies on population health. CA-A Cancer J Clin. (2024) 74:224–6. doi: 10.3322/caac.21838 38572764

[3] Mármol I Sánchez-De-Diego C Dieste AP Cerrada E Yoldi M . Colorectal carcinoma: A general overview and future perspectives in colorectal cancer. Int J Mol Sci. (2017) 18:197–1. doi: 10.3390/ijms18010197 28106826 PMC5297828

[4] Manca P Corti F Intini R Mazzoli G Miceli R Germani M . Tumour mutational burden as a biomarker in patients with mismatch repair deficient/microsatellite instability- high metastatic colorectal cancer treated with immune checkpoint inhibitors. Eur J Cancer. (2023) 187:15–24. doi: 10.1016/j.ejca.2023.03.029 37099945

[5] Fridman WH Pages F Sautes-Fridman C Galon J . The immune contexture in human tumours: impact on clinical outcome. Nat Rev Cancer. (2012) 12:298–306. doi: 10.1038/nrc3245 22419253

[6] Li JY Chen YP Li YQ Liu N Ma J . Chemotherapeutic and targeted agents can modulate the tumor microenvironment and increase the efficacy of immune checkpoint blockades. Mol Cancer. (2021) 20:27–1. doi: 10.1186/s12943-021-01317-7 33541368 PMC7863268

[7] Wei J Li WK Zhang PF Guo FK Liu M . Current trends in sensitizing immune checkpoint inhibitors for cancer treatment. Mol Cancer. (2024) 23:271–1. doi: 10.1186/s12943-024-02179-5 39725966 PMC11670468

[8] Kim SI Cassella CR Byrne KT . Tumor burden and immunotherapy: impact on immune infiltration and therapeutic outcomes. Front Immunol. (2021) 11. doi: 10.3389/fimmu.2020.629722 33597954 PMC7882695

[9] Sharma P Hu-Lieskovan S Wargo JA Ribas A . Primary, adaptive, and acquired resistance to cancer immunotherapy. Cell. (2017) 168:707–23. doi: 10.1016/j.cell.2017.01.017 28187290 PMC5391692

[10] Mohanty I Allaband C Mannochio-Russo H El Abiead Y Hagey LR Knight R . The changing metabolic landscape of bile acids-keys to metabolism and immune regulation. Nat Rev Gastroenterol Hepatol. (2024) 21:493–516. doi: 10.1038/s41575-024-00914-3 38575682 PMC12248421

[11] Caliceti C Punzo A Silla A Simoni P Roda G . New insights into bile acids related signaling pathways in the onset of colorectal cancer. Nutrients. (2022) 14:2964–14. doi: 10.3390/nu14142964 35889921 PMC9317521

[12] Joyce SA Gahan C . Disease-associated changes in bile acid profiles and links to altered gut microbiota. Dig Dis. (2017) 35:169–77. doi: 10.1159/000450907 28249284

[13] Rezen T Rozman D Kovács T Kovács P Sipos A Bai P . The role of bile acids in carcinogenesis. Cell And Mol Life Sci. (2022) 79:359–69. doi: 10.1007/s00018-022-04278-2 35429253 PMC9013344

[14] Liu YJ Zhang S. A Zhou W. J Hu D Xu H. C . Secondary bile acids and tumorigenesis in colorectal cancer. Front In Oncol. (2022) 12. doi: 10.3389/fonc.2022.813745 35574393 PMC9097900

[15] Dermadi D Valo S Ollila S Soliymani R Sipari N Pussila M . western diet deregulates bile acid homeostasis, cell proliferation, and tumorigenesis in colon. Cancer Res. (2017) 77:3352–63. doi: 10.1158/0008-5472.CAN-16-2860 28416481

[16] Chen XZ Ma Z. Y Yi Z. Q Wu E. Q Shang Z. Y Tuo B . The effects of metabolism on the immune microenvironment in colorectal cancer. Cell Death Discov. (2024) 10:118–1. doi: 10.1038/s41420-024-01865-z 38453888 PMC10920911

[17] Shahin M Patra S Purkait S Kar M Das Majumdar SK Mishra TS . PD-L1 expression in colorectal carcinoma correlates with the immune microenvironment. J GI Cancer. (2024) 55:940–9. doi: 10.1007/s12029-024-01049-z 38530597

[18] Wang HF Li Z Ou SW Song YN Luo KJ Guan ZL . Tumor microenvironment heterogeneity-based score system predicts clinical prognosis and response to immune checkpoint blockade in multiple colorectal cancer cohorts. Front In Mol Biosci. (2022) 9. doi: 10.3389/fmolb.2022.884839 35836930 PMC9274205

[19] Liu ZQ Liu ZQ Guo YX Yang XX Chen C Fan DD Wu XK . Immune landscape refines the classification of colorectal cancer with heterogeneous prognosis, tumor microenvironment and distinct sensitivity to frontline therapies. Front Cell Dev Biol. (2022) 9. doi: 10.3389/fcell.2021.784199 35083217 PMC8784608

[20] Chen YP Zheng X Wu CP . The role of the tumor microenvironment and treatment strategies in colorectal cancer. Front Immunol. (2021) 12. doi: 10.3389/fimmu.2021.792691 34925375 PMC8674693

[21] Jia W Xie GX Jia WP . Bile acid-microbiota crosstalk in gastrointestinal inflammation and carcinogenesis. Nat Rev Gastroenterol Hepatol. (2018) 15:111–28. doi: 10.1038/nrgastro.2017.119 29018272 PMC5899973

[22] Zhou XW Qiao KA Wu HM Zhang YY . The impact of food additives on the abundance and composition of gut microbiota. Molecules. (2023) 28:631–2. doi: 10.3390/molecules28020631 36677689 PMC9864936

[23] Liu JF Lichtenberg T Hoadley KA Poisson LM Lazar AJ Cherniack AD . An integrated TCGA pan-cancer clinical data resource to drive high-quality survival outcome analytics. Cell. (2018) 173:400. doi: 10.1016/j.cell.2018.02.052 29625055 PMC6066282

[24] Liberzon A Subramanian A Pinchback R Thorvaldsdóttir H Tamayo P . Molecular signatures database (MSigDB) 3. 0. Bioinformatics. (2011) 27:1739–40. doi: 10.1093/bioinformatics/btr260 21546393 PMC3106198

[25] Wilkerson MD Hayes DN . ConsensusClusterPlus: a class discovery tool with confidence assessments and item tracking. Bioinformatics. (2010) 26:1572–3. doi: 10.1093/bioinformatics/btq170 20427518 PMC2881355

[26] Carbon S Douglass E Dunn N Good B Harris NL Lewis SE . The Gene Ontology Resource: 20 years and still GOing strong. Nucleic Acids Res. (2019) 47:D330–8. doi: 10.1093/nar/gky1055 30395331 PMC6323945

[27] Kanehisa M Furumichi M Tanabe M Sato Y Morishima K . KEGG: new perspectives on genomes, pathways, diseases and drugs. Nucleic Acids Res. (2017) 45:D353–61. doi: 10.1093/nar/gkw1092 27899662 PMC5210567

[28] Szklarczyk D Gable AL Lyon D Junge A Wyder S Huerta-Cepas J . STRING v11: protein-protein association networks with increased coverage, supporting functional discovery in genome-wide experimental datasets. Nucleic Acids Res. (2019) 47:D607–13. doi: 10.1093/nar/gky1131 30476243 PMC6323986

[29] Jiang P Gu SQ Pan D Fu JX Sahu A Hu XH . Signatures of T cell dysfunction and exclusion predict cancer immunotherapy response. Nat Med. (2018) 24:1550. doi: 10.1038/s41591-018-0136-1 30127393 PMC6487502

[30] Kaiser AD Schuster K Gadiot J Borkner L Daebritz H Schmitt C . Reduced tumor-antigen density leads to PD-1/PD-L1-mediated impairment of partially exhausted CD8+T cells. Eur J Immunol. (2012) 42:662–71. doi: 10.1002/eji.201141931 22144176

[31] Shen RF Huang Y Kong DY Ma WH Liu J Zhang HZ . Spatial distribution pattern of immune cells is associated with patient prognosis in colorectal cancer. J Of Trans Med. (2024) 22:606–1. doi: 10.1186/s12967-024-05418-x 38951801 PMC11218284

[32] Sun LC Zhang HF Gao P . Metabolic reprogramming and epigenetic modifications on the path to cancer. Protein Cell. (2022) 13:877–919. doi: 10.1007/s13238-021-00846-7 34050894 PMC9243210

[33] He FF Li YM . Role of gut microbiota in the development of insulin resistance and the mechanism underlying polycystic ovary syndrome: a review. J Ovarian Res. (2020) 13:73–1. doi: 10.1186/s13048-020-00670-3 32552864 PMC7301991

[34] Court MH Hazarika S Krishnaswamy S Finel M Williams JA . Novel polymorphic human UDP-glucuronosyltransferase 2A3: Cloning, functional characterization of enzyme variants, comparative tissue expression, and gene induction. Mol Pharmacol. (2008) 74:744–54. doi: 10.1124/mol.108.045500 18523138 PMC2574548

[35] Sneitz N Court MH Zhang XL Laajanen K Yee KK Dalton P . Human UDP-glucuronosyltransferase UGT2A2: cDNA construction, expression, and functional characterization in comparison with UGT2A1 and UGT2A3. Pharmcog Genomics. (2009) 19:923–34. doi: 10.1097/FPC.0b013e3283330767 19858781 PMC2928392

[36] Chen XB Du P She JJ Cao L Li YC . Loss of ZG16 is regulated by miR-196a and contributes to stemness and progression of colorectal cancer. Oncotarget. (2016) 7:86695–703. doi: 10.18632/oncotarget.13435 27880730 PMC5349946

[37] Wu HD Zhong WL Zhang RH Ding YP Qu CH Lai KG . G-quadruplex-enhanced circular single-stranded DNA (G4-CSSD) adsorption of miRNA to inhibit colon cancer progression. Cancer Med. (2023) 12:9774–87. doi: 10.1002/cam4.5721 36855796 PMC10166891

[38] Gotoh-Saito S Abe T Furukawa Y Oda S Yokoi T Finel M . Characterization of human UGT2A3 expression using a prepared specific antibody against UGT2A3. Drug Metab Pharmacokinet. (2019) 34:280–6. doi: 10.1016/j.dmpk.2019.05.001 31262603

[39] Wei ZT Li F Pi GF . Association between gut microbiota and osteoarthritis: A review of evidence for potential mechanisms and therapeutics. Front Cell Infect Microbiol. (2022) 12. doi: 10.3389/fcimb.2022.812596 35372125 PMC8966131

