# Correction: Integrative subtyping by bile acid metabolism identifies *CLCA1*/*UGT2A3*/*ZG16* as markers of immune dysfunction and poor prognosis in colorectal cancer

**DOI:** 10.3389/fonc.2026.1964466

**Published:** 2026-08-27

**Authors:** Li Feng, Min Wang, Xin Li, Long Wu, DeXin Gu, Bin Zhang, Peng Zheng, Qifeng Yang, Ke Wang, Gang Mao

**Affiliations:** 1 Department of General Surgery, Hospital of Chengdu University of Traditional Chinese Medicine, Chengdu, China; 2 Department of Plastic and Aesthetic Surgery, Hospital of Chengdu University of Traditional Chinese Medicine, Chengdu, China; 3 Department of Vascular Surgery, Hospital of Chengdu University of Traditional Chinese Medicine, Chengdu, China; 4 Gastroenterology Department, Hospital of Chengdu University of Traditional Chinese Medicine, Chengdu, China

**Keywords:** bile acid metabolism, CLCA1, colorectal cancer, tumor immune microenvironment, UGT2A3, ZG16

An incorrect **Funding** statement was provided. The correct funder is Sichuan Science and Technology Program(Grant Number: 2024NSFSC0718). The correct **Funding** statement reads:

The author(s) declared that financial support was received for this work and/or its publication. This research was funded by Sichuan Science and Technology Program, with the project number: 2024NSFSC0718.

The original version of this article has been updated.

