# Supplementary figures and images for: Integrative subtyping by bile acid metabolism identifies CLCA1/UGT2A3/ZG16 as markers of immune dysfunction and poor prognosis in colorectal cancer
Source: Front Oncol. 2026 Jan 12;15:1739534. doi: 10.3389/fonc.2025.1739534 (PMC12832251; doi:10.3389/fonc.2025.1739534)

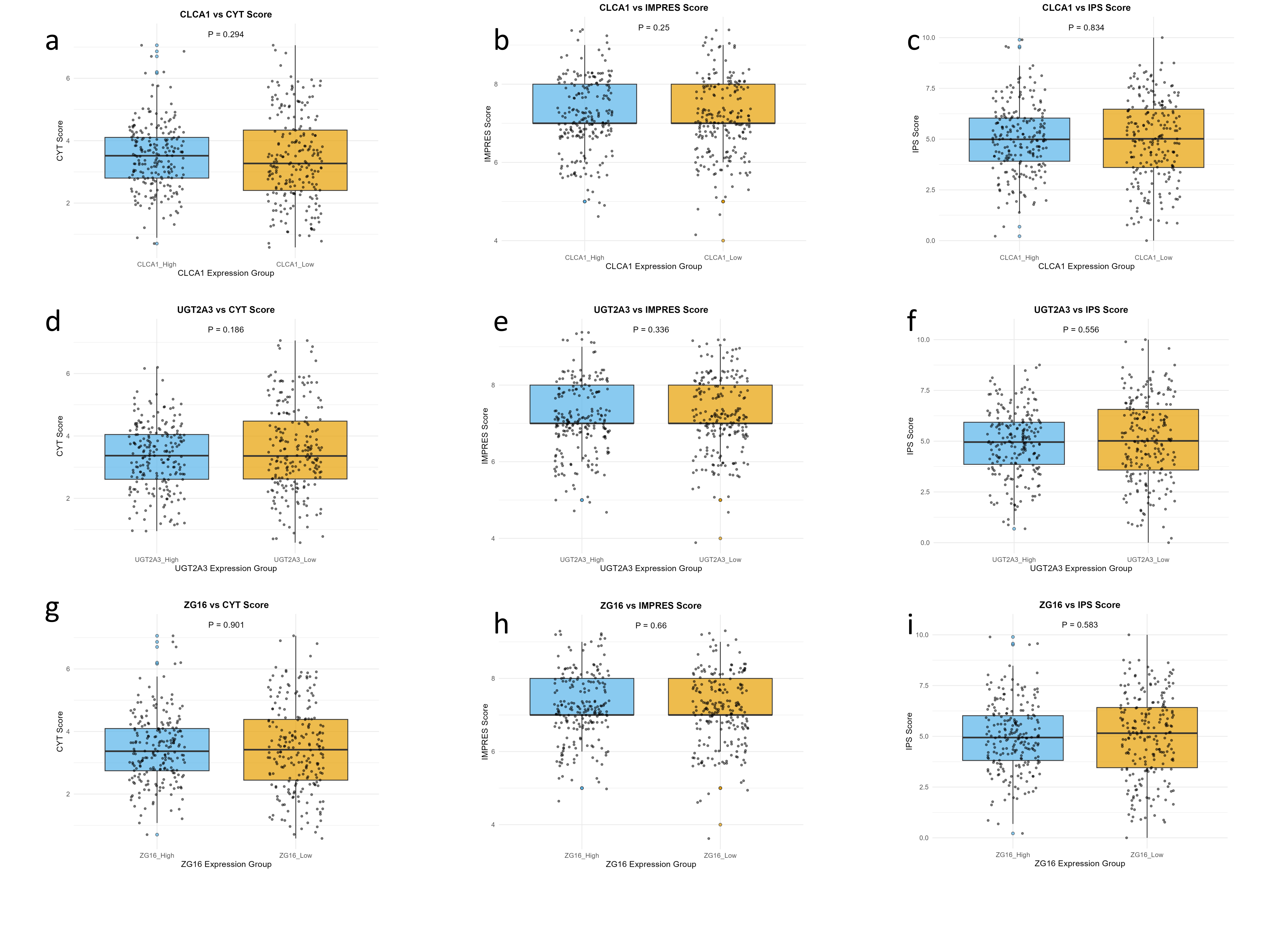

Supplement: Supplementary file 3 [file Image3.jpeg]
